# Recent Advancements towards Full-System Microfluidics

**DOI:** 10.3390/s17081707

**Published:** 2017-07-25

**Authors:** Amine Miled, Jesse Greener

**Affiliations:** 1 Electrical and Computer Engineering Department, Faculty of Sciences and Engineering, Université Laval, Quebec City, QC G1V 0A6, Canada; amine.miled@gel.ulaval.ca; 2 Department of Chemistry, Faculty of Sciences and Engineering, Université Laval, Quebec City, QC G1V 0A6, Canada

**Keywords:** microfluidic integration, microfabrication, microfluidics, MEMS, sensors, actuators, bio-microfluidics, point of care

## Abstract

Microfluidics is quickly becoming a key technology in an expanding range of fields, such as medical sciences, biosensing, bioactuation, chemical synthesis, and more. This is helping its transformation from a promising R&D tool to commercially viable technology. Fuelling this expansion is the intensified focus on automation and enhanced functionality through integration of complex electrical control, mechanical properties, in situ sensing and flow control. Here we highlight recent contributions to the Sensors Special Issue series called “Microfluidics-Based Microsystem Integration Research” under the following categories: (i) Device fabrication to support complex functionality; (ii) New methods for flow control and mixing; (iii) Towards routine analysis and point of care applications; (iv) In situ characterization; and (v) Plug and play microfluidics.

## 1. Introduction

Following the demonstration of the straightforward use of polydimethyl siloxane (PDMS) as a casting material for fabrication of microfluidic devices against photolithography moulds, there has been 20 years of intense work involving researchers from almost every scientific sub-domain [1,2,3,4]. During this time, the research community has learned how to take advantage of special liquid properties in microfluidic channels, which include dominance of surface and interfacial tensions and capillary forces as well as the lack of turbulence. These factors have enabled a number of new areas including chemical and materials synthesis [5], diagnostics for low-resource or remote settings [6,7], rapid processing and assaying biofluids [8], bioactuation [9], development of more physiologically relevant in vitro models and drug discovery [10,11,12,13], and more. Past and future growth of new applications and research relevance has been aided by strong focus on engineering and technical advancements. Microfluidic R&D multidisciplinarity and the constant stream of new applications is a source of inspiration for the community.

The transformation from an enabling R&D tool to commercially viable technology is rapidly underway. Currently, microfluidics is poised to become a key technology for a wide range of commercial and research technologies. Market analysis predicts sustained growth of nearly 20% with the absolute size expected to reach nearly $10 B by 2021. Now that the basic rules have been described and obvious applications have been addressed, an important new area for development is toward complex functionality via integrated, system-level approaches. Successful technologies using microfluidic elements have to be multifunctional, portable and easy to use. Fuelling this expansion is the intensified focus on automation and enhanced functionality via complex electrical components with sensing and flow control elements into robust channels with arbitrary geometries. To this end, we highlight recent contributions to the Sensors Special Issue called “Microfluidics-Based Microsystem Integration Research” under the following categories: (i) Device fabrication to support complex functionality; (ii) New methods for flow control and mixing devices; (iii) Towards routine analysis and point of care applications; (iv) In situ characterization; and (v) Plug and play microfluidics.

## 2. Device Fabrication to Support Complex Functionality

Along with low-cost and speed, new fabrication methods are needed to enhance device versatility. Today, most microfluidic research is still conducted using the popular elastomeric prototyping material PDMS. However, moving toward more robust, commercializable devices, especially those for biological work, will require different materials [14]. Thermoplastics are poised to accelerate next phase microfluidic devices due to their range of application-selective material properties and bonding methodologies. Related to the potential for supporting integrated systems, their physical robustness enables the integration of embedded probes, sensors and valves as well as reliable world-to-chip fluidic connections, all in a robust system that can operate for long times even under high pressures [15,16,17,18]. It can also solve the problem of PDMS permeability to small molecules, such as CO_2_ and O_2_. One approach to prototyping 3D structures via templating techniques, such as embossing or injection moulding, is to place emphasis on fabrication of low-cost templates with pre-designed features according to application requirements [19]. In their contribution to this special issue, the Greener and Kumacheva research groups teamed up with a Canadian microfluidic fabrication company, FlowJEM, approach the problem from a different direction. Here they developed a new one-step fabrication method for creating three-dimensional features in thermoplastic microchannels using a photo-lithography based (2D) embossing stamp [20]. With a little-used variation to hot embossing, called hot intrusion embossing, or partial embossing [21] they could create thermoplastic microfluidic channels with integrated features with controllable dimensions. To showcase the approach as a one-step step method for rapid fabrication of complex, robust, microfluidic platforms with integrated multi-functional elements, a microfluidic device was fabricated with an integrated three-dimensional mixer and a multi-focal length lens array. Other demonstrated in-channel features with controllable dimensions included posts, microlenses, walls, steps, tapered features and three-dimensional serpentine microchannels. The approach was applicable for a range of thermoplastics demonstrating the potential for wide application.

The proper sealing of microfluidic systems is a crucial hurdle for thermoplastic devices [22]. This is especially true for thermoplastic microfluidic systems with integrated electrodes due to the potential for typically aggressive bonding conditions to affect their performance. To date, the most common approaches include sealing via solvent bonding [23], laser welding [24], adhesive bonding [25], and thermal bonding [26,27]. To date, research is actively underway to develop methods which are both effective and compatible with current production standards. In the recent paper from the Matteucci group, a rigorous comparison of sealing via ultrasonic welding and thermal bonding [28]. The authors compared electrochemical performance following the bonding of injection moulded microfluidic chips with embedded thin-film gold electrodes. The work showed that bonding by ultrasonic wielding yielded chips with superior electrochemical performance compared to thermal bonding. In particular, yield was near 100% and variation in charge transfer resistance and double layer capacitance was low. The key to their success was low surface roughness during their injection moulding step [29], which enabled the use of lower energies during bonding. The authors demonstrated the potential for their approach to advance research, commercialization and education by mass-producing devices used by non-experts to perform and reproduce results of electrochemical measurement of yeast redox activity and the detection of dopamine as part of a summer school program.

## 3. New Methods and Techniques for Flow Control and Mixing

A recent explosion in affordable 3D printing technologies is having an impressive impact in the fabricated consumer product markets. With improved resolution, the microfluidic community has begun to embrace the technology [30]. The Lo group contributed to this special issue with a paper which used a high resolution variant of 3D printing, based on digital light processing (DLP) to fabricate a Tesla turbine which has applications in both power generation and fluid flow [31]. The team’s goal was to overcome the most important hurdle for the device’s real-world implementation—to maintain laminar flow between rotor disks—by scaling to the microfluidic flow regime. Based on DLP-based 3D printing resolution (approx. 40 µm laterally and 30 µm vertically), the resulting miniaturized Tesla turbine pump was characterized by low Reynolds number pumping (Re = 1000) at a flow rate of up to 12.6 mL/min and 1200 rpm. The pump was demonstrated by driving a microfluidic mixer network to generate microfluidic gradient. The miniaturized Tesla turbine pump has the potential to become an essential element in microfluidic system-level platforms.

Among new materials and their uses in microfluidic devices, integrated nanofibres can achieve interesting applications due to their high surface-areas and easy-to-functionalize surfaces. To date they have been demonstrated effective filters, concentrators and scaffolds within microfluidic devices. In this special issue, the Baeumner group demonstrated the use of electrospun PVA nanofibre mats to achieve passive mixing within thermoplastic microfluidic systems [32]. It was shown that the degree of mixing achieved in the downstream portion of Y-channels was most significant for fine nanofibres (450–550 nm diameter). The mixing was comparable to, or better than many reported passive mixers. The team notes that, in addition to mixing, the ability to functionalize nanofibers makes the approach a promising multi-functionality component within lab-on-a-chip devices.

Martínez-López and co-workers demonstrate the low-cost microfabrication approach called xurography—essentially a CNC knife plotter—as a rapid fabrication method for asymmetric split and recombine micromixing devices [33]. The team demonstrated that the technique resulted in structures with less than 8% absolute dimensional error. Numerical and experimental evaluation at the outlet determined mixing of up to 40%. The paper presents an encouraging approach for further research using xurography for micromixers as well as other functional elements in the future.

## 4. Towards Routine Analysis and Point of Care Applications

Building on their former work of creating nanostructured surfaces by induced folding in shape-memory polymer shrinking surfaces [34] integrated into microfluidic devices [35], the group of Moran-Mirabel demonstrated a benchtop fabrication method of a microfluidic electrochemical cell sensor based on 3D gold and platinum electrodes [36]. The device was sensitive, transparent and—importantly—flexible with shape memory. A key advancement was the PDMS to PDMS-structured electrode bonding protocol to fabricate the μF chip was optimized and found to have sufficient bond strength to withstand up to 100 mL/min flow rates. Using cyclic voltammetry applied to the structured electrodes, the authors used the device to correlate reduced charge transfer from a redox solution in the presence of murine 3T3 fibroblasts to detect as few as 24 cells. According to the authors, the combination of easy, low-cost fabrication for the demonstrated LoC system brings bedside point of care diagnostics and personalized medicine one step closer to being realized.

In addition to in situ electrical and electrochemical detection, embedded electrodes can also be used for the manipulation of colloidal matter and biological samples. Dielectrophorisis, for example, has been used in microchannels as a passive technique for sorting inert and biological particles, with potential for use in point of care applications [37,38,39]. As Páez-Avilés and co-workers demonstrate the combination of dielectrophoresis and impedance analysis in microfluidic format as a tool for enhanced bacterial detection [40]. The combination of dielectrophoresis and impedance can reduces measurement time and enhance sensitivity and selectivity, while microfluidics can help with simplicity of use and automation. The authors also discuss hurdles to overcome for sufficient impact in areas such as medicine, public health, agricultural, food control and environmental areas. 

Detection of cells and bacteria can also be conducted using optical methods, taking advantage of transparency of microfluidic devices. Typical measurements of optical density are used to monitor the growth rate of a suspended bacterial sample. However, this is not simple to do in low-path length environments of microchannels [41]. The Jeon group recently reported a system, for automated detection of bacteria using a camera that can observe blurring of a striped target pattern in a microchannel as planktonic bacteria concentrations increase [42]. The approach yields quantifiable results by using a fast Fourier transform conversion to observe a decrease in the high-frequency region of the marker image. The method was applied for detection of different bacterial types and fluidic device designs. The authors propose that the system can automate biological experiments, including antibiotics susceptibility test or toxicity measurements as well as a component for point-of-care devices. On the other hand, a paper from the Huang and co-workers demonstrates an approach for cell counting in microfluidic cytometer without resolving power of expensive optics [43]. A lensless system monitored peaks and valleys in overall light reaching a CMOS camera due to passing red blood cells. The authors tested different machine learning algorithms yielding results that compared well to commercial flow cytometers. The reduction in cost using a microfluidic approach has the potential to enable cytometry as a point of care technology.

A serious drawback for traditional microfluidic systems is the need for experienced users and the complexity of a typical setup, which includes tubing, valves and pumps. In their recent paper to this special issue, the Guan group presented an efficient high-throughput drug screening platform capable of quantitative combinatorial assays based on an arbitrarily accessible 3D microfluidic device [44]. The device featured automatic and simultaneous reagent loading and aliquoting tasks and performing multistep assays with arbitrary sequences and freedom from regular fluid handling systems, making it easy to operate; ideal for routine high-throughput drug screening outside traditional microfluidic labs. The device was benchmarked against a traditional microtiter plate-based method and has the potential for wide application in various combinatorial assays beyond high-throughput drug screening.

## 5. In Situ Characterization

Traditionally, most in situ characterisation in microchannels has been conducted by optical microscopes. However, sustained expansion requires a more diverse range of techniques. Challenges span the analytical and microfabrication domains, as sensitive sensors should become miniaturized and integrated into the microchannel environment. Publications in this special issue address this need. 

A novel microfluidic flow rate detection method based on surface plasmon resonance (SPR) temperature imaging is proposed. Wang and co-workers use spatially-resolved SPR imaging of the flow induced temperature variations as the basis for measurements of flow rates inside microchannels [45]. Using theoretical simulations and experimental analysis, the team demonstrated the proof of concept and determined range of usability between tens to hundreds of μL/min. Thanks to the wide application of SPR to bio-chemical microfluidic analysis the tool has the potential for multi-modal applications in integrated micro analytical systems.

The use of microfluidics environmental sensors is on the rise. For example, detection and quantification of industrial emissions is essential to protect workers and the environment close to and within factories. In work contributed by Lim and co-workers, different flexible ring resonator detectors were fabricated in microchanels for remote ethanol sensor applications. In the first, detectors of periodic split-ring-cross resonators were fabricated by simple inkjet printing of silver nanoparticle inks on paper [46]. The device’s performance was demonstrated by simulation and experiments, with a linear shift in resonance frequency over a wide range of ethanol concentrations. In the second, a complementary frequency-switchable split-ring resonator was shown to successfully detect 10% ethanol levels [47]. The third approach used a quarter mode substrate-integrated waveguide with a liquid metal alloy band-pass filter, which extended their complementary split ring resonator capabilities [48].

## 6. Bringing It All Together: Plug and Play Microfluidics

Full-blown microfluidic systems are on the horizon. To date plug and play (modular) concept for microfluidics has focused on fluidic connections [49,50,51] or reversibly integrated probes [18]. In this special issue, the Miled group builds on their previously developed concept of microfluidic modules for fluid actuation and electrode-based sensing and manipulations techniques via potentiometric, capacitive measurements and dielectrophorisis [13,39,52]. Here, they bring the concept to a new level by demonstrating modular components for a plug-and-play lab on a chip system for a potential system to treat neurodegenerative diseases [53]. Separate modules were devised for fluid control, noisome generation, a previously developed electrochemical imaging sensors system [54] and control wireless communication system control for implant applications. The modular approach was adopted for enabling measurements, drug delivery or both simultaneously. The platform represents a crucial step toward an “intelligent” drug delivery system based on a feedback loop to monitor drug delivery. In addition, it perfectly summarizes where we think highly integrated microfluidic systems are going in the future.

## 7. Conclusions

Microfluidics is an emerging and active research field. Increasingly, the point of contact between disciplines is at the application level. New developments to realize highly integrated microfluidic systems continue to require a broad, interdisciplinary approach. The Special Issue “Microfluidics-Based Microsystem Integration Research” highlights some of the exciting developments in this area, which are propelling microfluidic systems into wider acceptance and new applications.
